# Sex Difference in the Association Between Regional Adipose Tissue and Left Ventricular Hypertrophy

**DOI:** 10.3390/jcm14072399

**Published:** 2025-03-31

**Authors:** In-Jeong Cho, Sang-Eun Lee, Wook-Bum Pyun

**Affiliations:** Division of Cardiology, Department of Internal Medicine, Ewha Womans University Seoul Hospital, Ewha Womans University College of Medicine, Seoul 07804, Republic of Korea; seleemd@ewha.ac.kr (S.-E.L.); pwb423@ewha.ac.kr (W.-B.P.)

**Keywords:** adipose tissue, left ventricular hypertrophy, sex characteristics

## Abstract

**Background:** Left ventricular hypertrophy (LVH) is a key predictor of cardiovascular disease. This study aimed to investigate the correlation between LVH and regional adipose tissue deposits, including visceral adipose tissue (VAT), subcutaneous adipose tissue (SAT), and epicardial adipose tissue (EAT), and sex-related differences in these correlations. **Methods:** A total of 416 individuals (median age 66 years, range 20–95), without structural heart disease or a history of hypertension and coronary artery disease, underwent echocardiography and abdominal computed tomography. Statistical analyses included logistic and linear regression to assess the associations between adipose tissue measures and LVH. **Results:** LVH was associated with older age, higher systolic blood pressure, and increased EAT thickness. EAT thickness was independently associated with LVH in women (OR 1.21, 95% CI 1.03–1.44, *p* = 0.024) but not in men. Scatter plot analysis revealed a positive correlation between EAT and left ventricular mass index (LVMI) in women (r = 0.366, *p* < 0.001) and a negative correlation in men (r = −0.153, *p* = 0.038). **Conclusions:** VAT and SAT showed no significant associations with LVMI or LVH in either sex. These findings suggest that EAT, but not VAT or SAT, is linked to cardiac remodeling in a sex-specific manner.

## 1. Introduction

Left ventricular hypertrophy (LVH) is linked to increased cardiovascular mortality in both hypertensive patients and normotensive individuals [1]. Furthermore, evidence suggests that elevated left ventricular (LV) mass, even below the diagnostic threshold for LVH, correlates with heightened cardiovascular mortality risk [2]. Hypertension is the leading cause of LVH, but age and obesity are additional contributing factors [3,4].

Obesity is characterized by abnormal or excessive fat accumulation, with the body mass index (BMI) commonly used as a diagnostic criterion [5,6,7]. However, BMI inadequately reflects the distribution of adipose tissue. Comprehensive evaluation of body adiposity requires analysis of regional fat deposits, such as visceral adipose tissue (VAT) and subcutaneous adipose tissue (SAT). Among these, epicardial adipose tissue (EAT), a unique form of VAT situated between the myocardium and the visceral pericardium, is particularly significant in cardiovascular disease [8]. EAT has been linked to conditions such as coronary artery disease, atrial fibrillation, and heart failure [9]. Furthermore, in heart failure with preserved ejection fraction (HFpEF), an increased volume of EAT correlates with structural and functional myocardial abnormalities and independently predicts poorer outcomes regardless of BMI [10]. Previous studies have demonstrated that individuals with LVH tend to exhibit increased EAT [11,12]. However, the role of regional adipose tissue, including EAT, SAT, and VAT, in the development of LVH has not been fully explored in the previous studies.

HFpEF is more prevalent among women than men, suggesting that sex plays a pivotal role in in disease evolution. Pathophysiologically, women are more likely to develop concentric LVH due to chronic hypertension and metabolic disorders, with LVH being a major contributor to HFpEF [13]. Recent research underscores the importance of VAT over BMI in evaluating the cardiovascular effects of obesity [14], while the roles of other ectopic adipose tissues are increasingly recognized. Our previous study identified EAT as the most significant adipose tissue associated with small LV chamber size, concentric remodeling, and diastolic dysfunction compared to BMI, VAT, or SAT [15]. Adipose tissue distribution demonstrates well-established sex-specific patterns, with VAT more commonly accumulated in men and SAT predominating in women [16]. While the data on EAT are not entirely consistent, several reports have indicated that EAT thickness tends to increase substantially in elderly women, suggesting a sex-specific difference [15,17,18]. However, the sex-specific relationship between LVH and EAT has yet to be thoroughly investigated.

This study aims to investigate the relationship between LVH and various regional adipose tissue deposits, including VAT, SAT, and EAT, to better understand the role of obesity and adiposity in LVH progression. Furthermore, given the sex differences in the epidemiology and pathophysiology of HFpEF, we explored potential sex-related variations in the correlation between LVH and adipose tissue distribution.

## 2. Methods

### 2.1. Study Population

We performed a retrospective study on individuals who completed both transthoracic echocardiography and abdominal computed tomography (CT) within a 30-day timeframe at a single center in Korea during the period from January to December 2020 [15]. The exclusion criteria for the study were as follows: reduced LV systolic fraction (LV ejection fraction [LVEF] < 50%), regional wall motion abnormalities, pericardial effusion, significant valvular heart disease, cardiomyopathy, right ventricular dilation or dysfunction, inadequate echocardiographic imaging for diastolic function assessment, atrial fibrillation, polycystic kidney disease, single kidney, end-stage kidney disease requiring dialysis, and a history of kidney transplantation. Medical records were reviewed to collect data on the presence and medication histories of hypertension, diabetes mellitus, dyslipidemia, and coronary artery disease. Individuals with a history of hypertension, use of anti-hypertensive medications, or coronary artery disease were excluded. Ultimately, 416 participants with no structural heart disease, preserved LV systolic function, and no history of hypertension or coronary artery disease were included in the analysis. Blood pressure measurements, including systolic blood pressure (SBP) and diastolic blood pressure (DBP), were recorded prior to the echocardiography. The study methods were performed in accordance with the Declaration of Helsinki. This study protocol was reviewed and approved by the Institutional Review Board of Ewha Womans University Seoul Hospital (approval number: SEUMC 2024-04-001). The requirement for informed consent was waived by the Institutional Review Board of Ewha Womans University Seoul Hospital, given the retrospective study design.

### 2.2. Imaging Tests

Cardiac structure and function were evaluated using two-dimensional and Doppler echocardiography in adherence to the guidelines established by the American Society of Echocardiography [19]. Measurements included LV end-diastolic dimension (LVEDD) and LV end-systolic dimension (LVESD), interventricular septal thickness (IVST), and posterior wall thickness (PWT) from two-dimensional images in the parasternal long-axis view [18]. LV volume measurements were based on tracings of the blood tissue interface in the apical four- and two-chamber views, and LVEF was determined from end-diastolic and end-systolic volumes. LV mass was calculated based on LV wall thickness and LVEDD using the recommended formula, LV mass (g) = 0.8{1.04[([LVEDD + IVST +PWT]3 − LVEDD3)]}+ 0.6 [20], with the LV mass index (LVMI) computed by normalizing the LV mass to the body surface area. LVH was defined as an LVMI greater than 115 g/m^2^ in men and greater than 95 g/m^2^ in women [19]. Relative wall thickness (RWT) was derived as twice the posterior wall thickness divided by LVEDD. Left atrial volume was assessed using the biplane area–length method, which involved tracings of the blood–tissue interface and left atrial lengths obtained from apical four- and two-chamber views. This volume was then indexed to the body surface area to calculate the left atrial volume index (LAVI) [19]. Mitral inflow parameters, including early diastolic (E) velocity and septal early diastolic mitral annular (e’) velocity, were measured using pulse-wave Doppler, and the E/e’ ratio was calculated as an indicator of LV filling pressure. EAT thickness was measured as the maximum thickness during end-systole. Measurements were taken perpendicularly to the right ventricular free wall from the aortic annulus in the parasternal long-axis view [21].

CT imaging was utilized to assess perirenal adipose tissue (PAT) and SAT thickness. PAT was measured as the vertical distance from the posterior renal capsule to the posterior abdominal wall at the level of the renal vein in both the right and left kidneys [22]. An average of the right and left PAT thicknesses was calculated and used as a surrogate marker for VAT thickness [15]. SAT thickness was defined as the distance from the skin to the underlying musculature on the same imaging plane used for measuring left PAT [23]. The average time interval between echocardiography and CT performance was 6.1 ± 7.2 days.

### 2.3. Statistical Analysis

Demographic characteristics were reported as percentages for categorical variables and as means with standard deviations for continuous variables. Comparisons between patient groups were performed using chi-square tests for categorical data and Student’s *t*-tests for continuous data. Pearson’s correlation coefficient was calculated to evaluate simple correlations. Logistic regression analysis was used to identify factors associated with LVH, with odds ratios (ORs) and 95% confidence intervals (CIs) presented. Linear regression analysis was conducted to examine independent associations between variables. Variables with a *p*-value < 0.2 in univariate analysis were included in multivariate analysis. A *p*-value < 0.05 was considered statistically significant.

## 3. Results

### 3.1. Study Population

Table 1 summarizes the baseline characteristics of the study population. There were 376 subjects without LVH and 40 subjects with LVH. Compared to individuals without LVH, those with LVH were older (73 ± 11 vs. 63 ± 16 years, *p* < 0.001) and exhibited higher SBP (132 ± 17 vs. 126 ± 18 mmHg, *p* = 0.013). No significant differences were observed in the prevalence of female sex, diabetes, or dyslipidemia between the groups. LVH subjects showed elevated values in LVEDD (48.9 ± 4.5 vs. 46.0 ± 4.1 mm, *p* < 0.001), LVESD (28.6 ± 3.8 vs. 26.6 ± 3.5 mm, *p* < 0.001), LVMI (117.3 ± 18.0 vs. 78.0 ± 14.6 g/m^2^, *p* < 0.001), RWT (0.40 ± 0.77 vs. 0.36 ± 0.05, *p* = 0.001), LAVI (35.7 ± 12.6 vs. 27.2 ± 7.2 mL/m^2^, *p* < 0.001), and E/e’ ratio (13.6 ± 4.4 vs. 10.3 ± 3.8, *p* < 0.001). In contrast, e’ velocity was lower in the LVH group (5.7 ± 1.5 vs. 7.7 ± 2.6 cm/s, *p* < 0.001). EAT thickness was greater in the LVH group (6.3 ± 3.4 vs. 4.4 ± 2.8 mm, *p* < 0.001), although no significant differences were observed in BMI, VAT thickness, or SAT thickness between the groups.

Sex-stratified analysis revealed that men had higher SBP and DBP than women, with a greater proportion of men presenting with a SBP ≥ 140 mmHg. Additionally, men demonstrated higher values for LVEDD, LVESD, LVMI, and RWT. Conversely, women exhibited higher E velocity and E/e’ ratio values. EAT and SAT thickness were also greater in women, while VAT thickness was higher in men.

### 3.2. Factors Associated with LVH

Table 2 demonstrates the associated factors with LVH. Univariate and multivariate logistic analyses were conducted in the overall study population to identify factors associated with LVH. Since men and women showed different associations, we also performed separate analyses for each sex. In the overall population, increased age, elevated SBP, and greater EAT thickness were associated with LVH in univariate analysis. However, after adjusting for age, sex, SBP, body mass index (BMI), and EAT thickness in the multivariable analysis, age (odds ratio [OR] 1.05, 95% confidence interval [CI] 1.01–1.08, *p* = 0.011) emerged as the sole significant factor associated with LVH.

**Figure 1:**
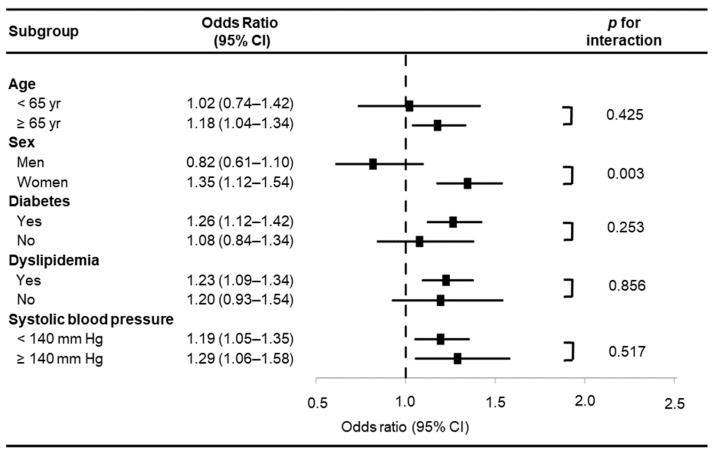
Estimated odds ratios for left ventricular hypertrophy in selected subgroups according to epicardial adipose tissue thickness. CI, confidence interval.

Figure 1 depicts the estimated ORs for LVH across subgroups categorized by EAT thickness. Subgroup analysis revealed a statistically significant interaction between EAT thickness and sex regarding LVH association (*p* for interaction = 0.003). In contrast, no significant interactions were observed between EAT thickness and other factors, such as age ≥ 65 years, diabetes mellitus, dyslipidemia, or SBP ≥ 140 mmHg. Given the significant interaction between EAT thickness and sex, a separate analysis was conducted to assess the differential impact of EAT thickness on LVH in men and women. The results, presented in Table 2, show that in men, increased age (OR 1.05, 95% CI 1.01–1.14, *p* = 0.027) was the only determinant for LVH after adjusting for BMI and EAT thickness. Conversely, in women, increased EAT thickness (OR 1.21, 95% CI 1.03–1.44, *p* = 0.024) was the sole independent factor associated with LVH, even after adjusting for age, SBP, BMI, and SAT thickness.

### 3.3. Correlations Among Age, BMI, Regional Adipose Tissues, and Echocardiographic Variables

**Figure 2:**
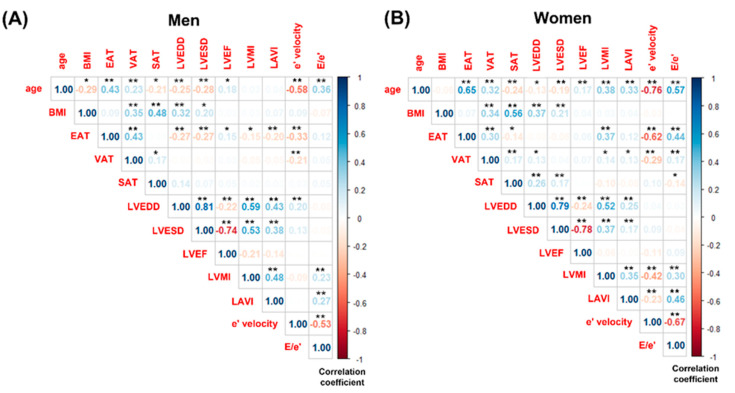
Grouped correlations among age, body mass index, regional adipose tissues, and echocardiographic variables. (**A**) Men and (**B**) Women. BMI, body mass index; EAT, epicardial adipose tissue; VAT, visceral adipose tissue; SAT, subcutaneous adipose tissue; LVEDD, left ventricular end-diastolic dimension; LVESD, left ventricular end-systolic dimension; LVEF, left ventricular ejection fraction; LVMI, left ventricular mass index; LAVI, left ventricular atrial volume index. Significance level: * = 0.05; ** = 0.01.

Figure 2 illustrates grouped correlations among age, BMI, various regional adipose tissues, and echocardiographic parameters. With increasing age, both EAT and VAT increased, while SAT decreased in both men and women. In men, BMI decreased with age (r = −0.294, *p* < 0.001), whereas no significant age-related change in BMI was observed in women. In both sexes, LV chamber size declined with age, accompanied by an increase in LVEF. Additionally, both genders showed a reduction in e’ velocity and an elevation in the E/e’ ratio with age. However, an increase in LVMI (r = 0.038, *p* < 0.001) and LAVI (r = 0.331, *p* < 0.001) was observed exclusively in women.

**Figure 3:**
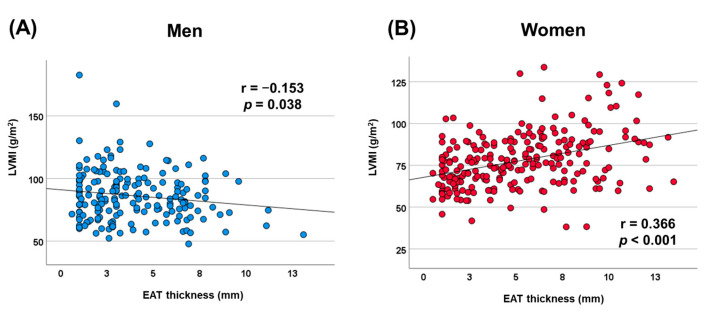
Scatterplot displaying the correlation between left ventricular mass index and epicardial adipose tissue thickness. (**A**) Men and (**B**) women. LVMI, left ventricular mass index; EAT, epicardial adipose tissue.

In men, no significant correlations were found between LVMI and BMI (r = 0.029, *p* = 0.692), VAT (r = −0.051, *p* = 0.496), or SAT (r = 0.023, *p* = 0.759). Conversely, in women, VAT demonstrated a significant correlation with LVMI (r = 0.136, *p* = 0.037), while BMI (r = 0.031, *p* = 0.636) and SAT (r = 0.126, *p* = 0.126) did not exhibit significant associations with LVMI. Figure 3 also displays scatter plots showing the correlation between LVMI and EAT thickness separately for men and women. In women, a significant positive correlation was observed between LVMI and EAT thickness (r = 0.366, *p* < 0.001). In contrast, a statistically significant negative correlation was found between LVMI and EAT thickness in men (r = −0.153, *p* = 0.038).

### 3.4. Factors Associated with EAT

To evaluate whether the factors associated with EAT differ by sex, we conducted linear regression analyses separately for men and women. In the univariate analysis, EAT was associated with age and VAT in men, while in women, EAT was linked to age, diabetes mellitus, SBP, VAT, and SAT.

In the multivariate analysis, after adjusting for diabetes mellitus, SBP, and SAT thickness, EAT in men remained significantly associated with age (beta = 0.354, *p* < 0.001) and VAT (beta = 0.344, *p* < 0.001). Conversely, in women, EAT was independently associated with age (beta = 0.565, *p* < 0.001), diabetes mellitus (beta = 0.178, *p* < 0.001), and SBP (beta = 0.015, *p* = 0.045) after adjusting for VAT and SAT in the multivariate model.

## 4. Discussion

The principal findings of the current study are that (1) a sex-specific difference was observed in the association between EAT thickness and LVH; (2) increased EAT thickness was significantly associated with the presence of LVH and higher LVMI in women, but these relationships were not evident in men; and (3) BMI, VAT, and SAT were not correlated with LVH or LVMI in either sex, underscoring the limited relevance of these measures in this context.

### 4.1. LVH, Obesity, and EAT

LVH is a strong predictor of cardiovascular disease, independent of traditional risk factors, and its regression is associated with improved cardiovascular outcomes [24,25,26]. The primary cause of LVH is hypertension, though age and obesity are also significant contributing factors [3,4]. Obesity, particularly when associated with increased VAT, is a major driver of hypertension, accounting for 65% to 75% of the risk for essential hypertension. In obese individuals, LVH and associated geometric changes are driven by cardiomyocyte hypertrophy, interstitial fat deposition, and triglyceride accumulation within myocardial contractile elements [27].

While obesity is typically classified using BMI, this metric does not fully capture the complexity of excess adiposity. Excess body fat is increasingly recognized as a heterogeneous condition, with individuals of similar BMI exhibiting varying risks for metabolic and cardiovascular diseases [28]. VAT is considered a more accurate reflection of cardiovascular risk compared to BMI and has a significant impact on cardiovascular outcomes [29].

EAT, a distinct form of VAT located within the heart, has emerged as a predictor of various cardiovascular diseases and heart failure [18]. While EAT probably has a function in cardiac homeostasis during health, it is thought that this function can be disrupted by accumulation and inflammation of EAT [18]. Since there is no basal layer between EAT and the myocardium, EAT may directly interact with, or infiltrate into the underlying myocardium [18]. Accumulation of EAT has consistently been associated with LVH, diastolic dysfunction, and atrial dilatation [30], which all are considered typical hallmarks of HFpEF. Although the precise mechanisms linking EAT to HFpEF remain unclear, two primary hypotheses have been proposed. The first is the infiltrative-lipotoxic hypothesis, suggesting that lipid infiltration and toxicity affect myocardial function [31]. The second is the pericardial restraint hypothesis, positing that EAT exerts mechanical compression on the heart, impairing function. Although obesity is currently defined by BMI, some individuals have a BMI in the obese range but do not exhibit increased cardiovascular risk, a condition referred to as metabolically healthy obesity [32,33]. These findings suggest that adipose tissue accumulation and distribution, rather than BMI alone, may play a more critical role in the pathogenesis of cardiovascular diseases. The impairment in myocardial function detected in individuals with obesity may be primarily related to the mechanical compression on cardiac chambers exerted by the combined action of abdominal adiposity and EAT [34]. The impairment in cardiac function caused by extrinsic compressive phenomena is more pronounced in individuals with a narrow antero-posterior thoracic diameter [34]. Taken together, regardless of the mechanism, EAT contributes to heart failure by promoting LVH, impaired LV diastolic function, and elevated filling pressures [18].

### 4.2. Sex Differences in LVH and EAT

There are notable sex differences in adipose tissue characteristics and fat distribution patterns. Men typically accumulate fat centrally and have larger visceral omental fat cells compared to women [16]. Clinical studies show conflicting data regarding the amount of EAT in men compared to women [17,35]. This discrepancy may in part be related to the post-menopausal status of women, as older women of >60 years have higher EAT volumes compared to younger women [17]. In our study, patients with an average age older than 60 years demonstrated higher EAT thickness and SAT thickness, but lower VAT thickness in women compared to men.

Interestingly, we found that there was a significant statistical interaction between EAT thickness and sex with regards to the association with LVH. A positive association between EAT thickness and both LVH and LVMI was observed only in women. In contrast, no such association was evident in men. This discrepancy may be explained by the differential metabolic roles of EAT in men and women. In men, EAT was primarily associated with VAT, while in women, EAT was related to metabolic factors such as diabetes mellitus, elevated SBP, and age. This suggests that in women, increased EAT thickness is more likely to develop alongside other metabolic components beyond simple obesity or VAT. Additionally, EAT thickness tends to increase rapidly in women after age 60, with values surpassing those observed in men [15,17]. Prior studies have reported that EAT is linked to poorer LV systolic and diastolic function in women but not in men [17]. Consistent with these findings, our study demonstrated a closer association between EAT and both LVMI and diastolic function in women, suggesting stronger interactions between EAT and myocardial structure and function in women compared to men.

The infiltrative-lipotoxic hypothesis offers a potential explanation for this phenomenon, proposing that during its pathological transformation, EAT infiltrates the myocardium, disrupting its ultrastructure and function, which may lead to LVH [18]. Ectopic fat deposition in the myocardium is closely linked to LVH and diastolic dysfunction, particularly in metabolic diseases [36,37]. Myocardial steatosis has been suggested as a potential mechanistic link between diastolic dysfunction and coronary microvascular dysfunction in women [36]. Furthermore, myocardial steatosis appears to increase with BMI and glucose impairment in women but not in men [38], which aligns with our observation that the association between EAT and LVH was more pronounced in women. Given that women are more prone to developing myocardial steatosis and LVH due to metabolic disorders, further research is needed to investigate whether myocardial steatosis is the key factor driving LVH in women with thick EAT. Additionally, as obesity is more strongly associated with HFpEF in women than in men [39], further exploration of this topic is warranted.

## 5. Limitations

The primary limitation of this study was its retrospective design. Nevertheless, thorough reviews of patient medical records and echocardiographic and CT images were performed to minimize potential bias. Another limitation was the measurement of adipose tissue thickness in a single plane, despite its three-dimensional distribution around organs. Although individuals with a history of diagnosed hypertension were excluded to reduce the confounding effects of blood pressure and antihypertensive medications, 24.3% of participants had a SBP of 140 mmHg or higher. Thus, the influence of elevated blood pressure could not be entirely excluded. Nevertheless, the influence of hypertension status on the sex-specific association between EAT and LVH—the primary finding of this study—appears minimal, given that no significant interaction was detected between SBP levels (<140 mmHg vs. ≥140 mmHg) and the EAT–LVH relationship, as demonstrated in Figure 1. Additionally, the average BMI of the study population was approximately 23 kg/m^2^, with a low proportion of obese individuals, even by Asian obesity classification standards. This limits the generalizability of the findings to populations with severe obesity.

## 6. Conclusions

EAT thickness demonstrated a strong association with LVH and elevated LVMI in women, whereas no such relationship was observed in men. This finding suggests a potential sex-specific interaction between regional adiposity and cardiac remodeling. In contrast, conventional adiposity measures such as BMI, VAT, and SAT were not significantly associated with LVH or LVMI in either sex. These results indicate that EAT evaluation may provide more informative insights into cardiovascular risk stratification than traditional anthropometric indices, particularly in women. However, as a hypothesis-generating study, our analysis does not clarify the underlying mechanisms driving these sex-specific associations. Further research is warranted to explore the relationship between LVH, HFpEF, and EAT, with particular attention paid to potential sex-related differences.

## Figures and Tables

**Table 1:** Study population.

Variable	LVH			Sex		
	No LVH (*n* = 376)	LVH (*n* = 40)	*p*-Value	Men (*n* = 183)	Women (*n* = 233)	*p*-Value
Age, years	63 ± 16	73 ± 11	<0.001	65 ± 15	63 ± 16	0.082
Women, *n* (%)	205 (54.5)	28 (70.0)	0.067	-	-	-
Diabetes mellitus, *n* (%)	56 (14.9)	6 (15.0)	0.999	33 (18.0)	29 (12.4)	0.112
Dyslipidemia, *n* (%)	53 (14.1)	6 (15.0)	0.814	28 (15.3)	31 (13.3)	0.562
SBP, mm Hg	126 ± 18	132 ± 17	0.013	128 ± 18	125 ± 18	0.039
DBP, mm Hg	74 ± 11	74 ± 10	0.399	75 ± 11	73 ± 11	0.040
SBP ≥ 140 mm Hg	90 (23.9)	11 (27.5)	0.698	55 (30.1)	46 (19.7)	0.015
BMI, kg/m^2^	23.4 ± 3.8	23.5 ± 3.6	0.436	22.9 ± 3.4	23.8 ± 3.9	0.008
Echocardiographic variable						
LVEDD, mm	46.0 ± 4.1	48.9 ± 4.5	<0.001	47.8 ± 4.3	45.1 ± 3.9	<0.001
LVESD, mm	26.6 ± 3.5	28.6 ± 3.8	<0.001	27.6 ± 3.6	26.1 ± 3.4	<0.001
LVEF, %	66.5 ± 5.3	65.6 ± 5.3	0.161	66.4 ± 5.1	66.4 ± 5.5	0.453
LVMI, g/m^2^	78.0 ± 14.6	117.3 ± 18.0	<0.001	86.3 ± 19.7	77.7 ± 16.4	<0.001
RWT	0.36 ± 0.05	0.40 ± 0.07	0.001	0.37 ± 0.05	0.36 ± 0.06	0.027
LAVI, mL/m^2^	27.2 ± 7.2	35.7 ± 12.6	<0.001	28.4 ± 8.1	27.7 ± 8.3	0.191
E velocity, cm/s	73.4 ± 18.9	73.3 ± 19.2	0.480	69.7 ± 19.3	76.3 ± 18.1	<0.001
e’ velocity, cm/s	7.7 ± 2.6	5.7 ± 1.5	<0.001	7.5 ± 2.3	7.6 ± 2.8	0.322
E/e’	10.3 ± 3.8	13.6 ± 4.4	<0.001	9.8 ± 3.2	11.2 ± 4.4	<0.001
EAT thickness, mm	4.4 ± 2.8	6.3 ± 3.4	<0.001	3.9 ± 2.5	5.2 ± 3.1	<0.001
CT variable						
Visceral adipose tissue						
Average PAT thickness, mm	9.0 ± 6.2	8.5 ± 4.4	0.299	10.5 ± 6.9	7.7 ± 5.0	<0.001
SAT thickness, mm	19.4 ± 8.9	19.6 ± 7.5	0.414	14.2 ± 5.9	23.5 ± 8.4	<0.001

LVH, left ventricular hypertrophy; SBP, systolic blood pressure; DBP, diastolic blood pressure; BMI, body mass index; LVEDD, left ventricular end-diastolic dimension; LVESD, left ventricular end-systolic dimension; LVEF, left ventricular ejection fraction; LVMI, left ventricular mass index, RWT, relative wall thickness; LAVI, left atrial volume index; CT, computed tomography, PAT, perirenal adipose tissue; SAT, subcutaneous adipose tissue.

**Table 2:** Associated factors for left ventricular hypertrophy in men and women.

Variable	Univariable		Multivariable	
	OR (95% CI)	*p*-Value	OR (95% CI)	*p*-Value
All patients (*n* = 416)				
Age, per year	1.05 (1.03–1.08)	<0.001	1.05 (1.01–1.08)	0.011
Women	1.97 (0.96–3.94)	0.065	1.89 (0.87–4.10)	0.105
Diabetes mellitus	1.01 (0.41–2.51)	0.986	-	-
Dyslipidemia	1.08 (0.43–2.69)	0.876	-	-
SBP, per mmHg	1.02 (1.00–1.04)	0.028	1.01 (0.99–1.03)	0.165
DBP, per mmHg	1.00 (0.97–1.03)	0.810	-	-
BMI, per kg/m^2^	1.01 (0.92–1.10)	0.873	1.01 (0.92–1.12)	0.794
EAT, per mm	1.22 (1.10–1.36)	<0.001	1.06 (0.93–1.22)	0.366
VAT, per mm	0.99 (0.93–1.04)	0.597	-	-
SAT, per mm	1.00 (0.97–1.04)	0.871	-	-
Men (*n* = 183)				
Age, per year	1.04 (0.99–1.09)	0.142	1.01 (1.01–1.14)	0.027
Diabetes mellitus	0.90 (0.19–4.33)	0.899	-	-
Dyslipidemia	1.12 (0.23–5.39)	0.892	-	-
SBP, per mmHg	1.01 (0.98–1.04)	0.590	-	-
DBP, per mmHg	1.00 (0.95–1.01)	0.978	-	-
BMI, per kg/m^2^	0.96 (0.81–1.15)	0.676	1.08 (0.88–1.33)	0.464
EAT, per mm	0.82 (0.61–1.10)	0.182	0.68 (0.47–1.00)	0.050
VAT, per mm	0.95 (0.85–1.05)	0.317	-	-
SAT, per mm	1.05 (0.95–1.12)	0.317	-	-
Women (*n* = 233)				
Age, per year	1.06 (1.03–1.10)	<0.001	1.03 (0.98–1.04)	0.223
Diabetes mellitus	1.20 (0.38–3.75)	0.754	-	-
Dyslipidemia	1.10 (0.35–3.41)	0.871	-	-
SBP, per mmHg	1.03 (1.01–1.05)	0.012	1.02 (0.99–1.04)	0.239
DBP, per mmHg	0.99 (0.96–1.04)	0.933	-	-
BMI, per kg/m^2^	1.01 (0.91–1.11)	0.864	1.03 (0.90–1.18)	0.657
EAT, per mm	1.35 (1.18–1.54)	<0.001	1.21 (1.03–1.44)	0.024
VAT, per mm	1.03 (0.96–1.11)	0.421	-	-
SAT, per mm	0.96 (0.92–1.01)	0.130	0.97 (0.91–1.04)	0.423

OR, odds ratio; CI, confidence interval; SBP, systolic blood pressure; DBP, diastolic blood pressure; BMI, body mass index; EAT, epicardial adipose tissue; VAT, visceral adipose tissue; SAT, subcutaneous adipose tissue.

## Data Availability

The datasets generated or analyzed during the current study are available from the corresponding author on reasonable request.

## Abbreviations

CI	confidence interval
CT	computed tomography
DBP	diastolic blood pressure
BMI	body mass index
EAT	epicardial adipose tissue
HFpEF	heart failure with preserved ejection fraction
IVST	interventricular septal thickness
LAVI	left atrial volume index
LV	left ventricular
LVEDD	left ventricular end-diastolic dimension
LVEF	left ventricular ejection fraction
LVESD	left ventricular end-systolic dimension
LVH	left ventricular hypertrophy
LVMI	left ventricular mass index
OR	odds ratio
PAT	perirenal adipose tissue
PWT	posterior wall thickness
RWT	relative wall thickness
SAT	subcutaneous adipose tissue
SBP	systolic blood pressure
VAT	visceral adipose tissue
